# Retroviral DNA—the silent winner: blood transfusion containing latent feline leukemia provirus causes infection and disease in naïve recipient cats

**DOI:** 10.1186/s12977-015-0231-z

**Published:** 2015-12-21

**Authors:** Stefanie Nesina, A. Katrin Helfer-Hungerbuehler, Barbara Riond, Felicitas S. Boretti, Barbara Willi, Marina L. Meli, Paula Grest, Regina Hofmann-Lehmann

**Affiliations:** Clinical Laboratory, Vetsuisse Faculty, University of Zurich, Zurich, Switzerland; Center for Clinical Studies, Vetsuisse Faculty, University of Zurich, Zurich, Switzerland; Clinic for Small Animal Internal Medicine, Vetsuisse Faculty, University of Zurich, Zurich, Switzerland; Institute of Veterinary Pathology, Vetsuisse Faculty, University of Zurich, Zurich, Switzerland

**Keywords:** Feline leukemia virus, Retrovirus, Latent infection, Proviral DNA, Provirus carrier, Infection risk, Blood transfusion, Transmission route, Reactivation of infection, Immune response

## Abstract

**Background:**

The feline leukemia virus (FeLV) is a gamma-retrovirus of domestic cats that was discovered half a century ago. Cats that are infected with FeLV may develop a progressive infection resulting in persistent viremia, immunodeficiency, tumors, anemia and death. A significant number of cats mount a protective immune response that suppresses viremia; these cats develop a regressive infection characterized by the absence of viral replication and the presence of low levels of proviral DNA. The biological importance of these latter provirus carriers is largely unknown.

**Results:**

Here, we demonstrate that ten cats that received a transfusion of blood from aviremic provirus carriers developed active FeLV infections, some with a progressive outcome and the development of fatal FeLV-associated disease. The infection outcome, disease spectrum and evolution into FeLV-C in one cat mirrored those of natural infection. Two cats developed persistent antigenemia; six cats were transiently antigenemic. Reactivation of infection occurred in some cats. One recipient developed non-regenerative anemia associated with FeLV-C, and four others developed a T-cell lymphoma, one with secondary lymphoblastic leukemia. Five of the ten recipient cats received provirus-positive aviremic blood, whereas the other five received provirus- and viral RNA-positive but aviremic blood. Notably, the cats that received blood containing only proviral DNA exhibited a later onset but graver outcome of FeLV infection than the cats that were transfused with blood containing proviral DNA and viral RNA. Leukocyte counts and cytokine analyses indicated that the immune system of the latter cats reacted quicker and more efficiently.

**Conclusions:**

Our results underline the biological and epidemiological relevance of FeLV provirus carriers and the risk of inadvertent FeLV transmission via blood transfusion and demonstrate the replication capacity of proviral DNA if uncontrolled by the immune system. Our results have implications not only for veterinary medicine, such as the requirement for testing blood donors and blood products for FeLV provirus by sensitive polymerase chain reaction, but are also of general interest by revealing the importance of latent retroviral DNA in infected hosts. When aiming to eliminate a retroviral infection from a population, provirus carriers must be considered.

**Electronic supplementary material:**

The online version of this article (doi:10.1186/s12977-015-0231-z) contains supplementary material, which is available to authorized users.

## Background

The feline leukemia virus (FeLV) is a retrovirus that occurs worldwide in domestic cats and some related small felids [1, 2]. FeLV infection can induce cytoproliferative (tumors) and cytosuppressive (immunodeficiency, anemia) diseases with fatal outcomes. FeLV isolates are categorized into several subgroups according to their viral receptor usage and interference capabilities. FeLV-A is the common subgroup, which is horizontally transmitted, highly contagious, but low in pathogenicity [1, 3]. FeLV-A infected cats may develop diseases, such as lymphoma mainly of T-cell origin. FeLV-B and FeLV-C arise within FeLV-A infected cats by recombination with endogenous FeLV-like sequences (enFeLV) and via mutations or insertions in the surface glycoprotein gene, respectively [4, 5]. FeLV-B is found in ~50 % of the FeLV-infected cats, whereas FeLV-C develops in only ~1 % of FeLV infections and is strongly associated with the development of non-regenerative anemia [3, 6, 7]. Furthermore, FeLV-T, a T-cell tropic cytopathic virus that causes lymphoid depletion and immunodeficiency, may develop from FeLV-A in infected cats via mutations and insertions in the surface glycoprotein gene [8]. Finally, most recently a FeLV-D subgroup was described; FeLV-D are recombinant viruses that arise after transduction of the envelope gene of feline endogenous gammaretrovirus, ERV-DC, into FeLV [9].

There are a variety of outcomes from FeLV infection, which are not only influenced by the strain, subtype and dose of the virus but also by different host factors, such as the age and immune system status of the infected cat [1]. The spectrum of different infection outcomes is classified according to results from FeLV antigen (p27) and nucleic acid detection and serology into abortive, regressive, progressive or atypical infections [10–13]. Detection of the p27 antigen is a parameter for viremia in most cats [14]. In addition, latent non-productive infection, characterized by the absence of viremia and the persistence of the virus in the bone marrow, can be identified in cats following regressive infection [15, 16]. Latent infection is detected by the prolonged cultivation of bone marrow cells in the presence of hydrocortisone [16] and usually resolves within a few months of exposure to FeLV but has been detected in some cats up to 30 months after infection [17]. Although persistently infected cats fail to develop a successful immune response to FeLV and succumb to FeLV-associated diseases, cats with regressive infections (previously also referred to as “recovered cats”) [18] mount a cellular and humoral immune response to FeLV and overcome viremia usually after a few weeks; however, these cats typically remain provirus-positive for the remainder of their lives [19]. The prevalence of provirus positive and p27-negative cats varies among different investigated populations and has been observed to be as high as 10 % [19, 20]. The clinical importance of the provirus positive status is not completely understood but may contribute to a long-lasting maintenance of protective immunity against FeLV [21]. However, reactivation of the infection with development of FeLV-associated diseases has been documented in many provirus positive cats [11, 15, 16, 22, 23].

FeLV is transmitted horizontally, mainly via saliva from persistently infected cats but also vertically from infected queens to their kittens. The virus is also detectable in feces, urine, milk and blood [24–28]. Iatrogenic transmission of FeLV may occur via the transfusion of blood from FeLV viremic (p27 antigen-positive) blood donors [29], and blood recipients may be particularly prone to infection and FeLV-associated disease due to pre-existing grave clinical conditions and possible immunosuppression. Common reasons for blood transfusion in cats include hemorrhages, lack of erythropoiesis and immune-mediated hemolytic anemia [30]. Blood banks for cats have been developed, and the demand for high-quality blood products has increased [30, 31]. The screening of blood donors and products for infectious diseases is of increasing importance. It is commonly recommended to test feline blood donors for FeLV infection by antigen detection [32]. So far, the risk of a FeLV transmission by transfusion of provirus positive blood has not been evaluated. However, intradermal inoculation of plasmid deoxyribonucleic acid (DNA) carrying the FeLV-A provirus (Rickard/FRA) has resulted in productive FeLV infection [33]. Therefore, we hypothesized that whole blood containing white blood cells carrying the FeLV provirus will transfer the infection and result in full-blown infection in recipient cats. The aim of the present study was thus to test whether FeLV infection can be transmitted via the transfusion of blood from a FeLV provirus positive and antigen negative blood donor to a naïve recipient cat.

## Results

### Transmission of FeLV by the transfusion of provirus-positive blood

To test whether FeLV infection can be transmitted by the transfusion of provirus positive blood, 15 specified pathogen-free (SPF) kittens were each transfused with 10 mL of blood. Five kittens in group A (P1–P5) received blood from a provirus-positive cat (DP), five kittens in group B (R1–R5) received blood from a provirus- and viral RNA-positive cat (DR), and five cats in the control group C (N1–N5) received naïve blood from an SPF cat (DN; Table 1). All three blood donor cats tested FeLV-negative in virus isolation and negative for the p27 antigen. Blood samples were collected from the recipients prior to (week 0) and in weekly intervals after the transfusion for 15 weeks. During and after the blood transfusion, no adverse reactions were observed in any of the recipients. According to the FeLV loads in the transfused blood, each cat in group A received a total of 1 × 10^4^ provirus copies, and each recipient in group B received 6.25 ×10^4^ provirus and 6.2 × 10^5^ viral RNA copies. The packed cell volume (PCV) of cat DR was 32 % at the time point of blood collection for transfusion; this value was used to calculate the viral RNA load per mL of whole blood as described in the “Methods” section.

**Table 1:** Outcome of FeLV infection in the two FeLV-exposed blood donors and the ten exposed recipient cats

	Group	Cat	Outcome of FeLV exposure	Health status long-term follow-up^a^
Blood donors	A	DP	Regressive infection, provirus-positive	Healthy
	B	DR	Regressive infection, viral plasma RNA- and provirus-positive	^†^Week 60: reactivation of infection, multicentric T-cell lymphoma^b^
Recipients	A	P1	Progressive infection, persistently p27-positive	^†^Week 31: non-regenerative anemia (FeLV-C)^b^
		P2	Progressive infection, persistently p27-positive	^†^Week 84: multicentric T-cell lymphoma^b,c^
		P3	Regressive infection, provirus-positive	Week 94: feline lower urinary tract disease (FLUTD)
		P4	Regressive infection, provirus-positive	Healthy
		P5	Regressive infection, provirus-positive	^†^Week 133: reactivation of infection, multicentric T-cell lymphoma^b^
	B	R1	Regressive infection, provirus-positive	^†^Week 26: reactivation of infection, multicentric T-cell lymphoma with secondary lymphoblastic leukemia^b^
		R2	Regressive infection, provirus-positive	Healthy
		R3	Regressive infection, provirus-positive	Healthy
		R4	Regressive infection, provirus-positive	^†^Week 28: multicentric T-cell lymphoma; no reactivation of the infection (p27-negative)^b^
		R5	Regressive infection, provirus-positive	Healthy

^†^euthanized/died

^a^Status observed until 149 weeks post transfusion

^b^Euthanized for humane reasons

^c^During follow-up study, without re-exposure to FeLV [35]

**Fig. 1:**
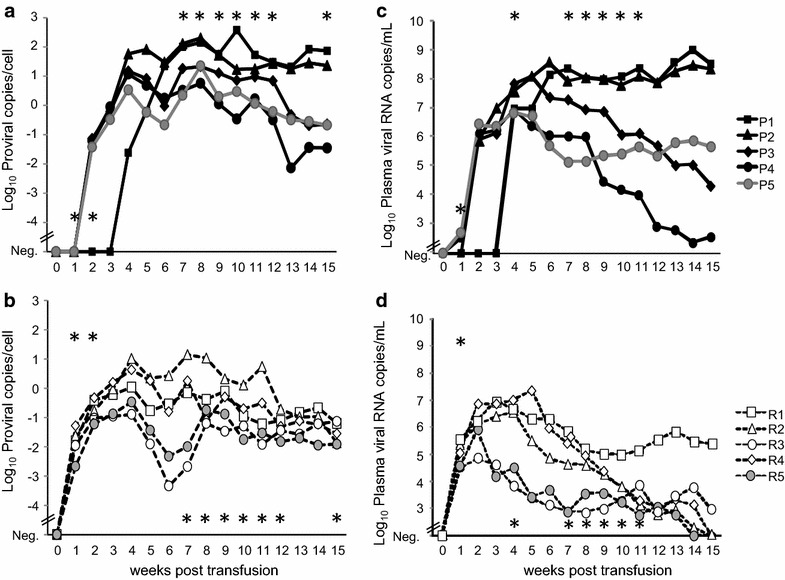
Proviral blood loads (**a**, **b**) and plasma viral RNA loads (**c**, **d**) of the five cats in group A (**a**, **c**) and the five cats in group B (**b**, **d**). The proviral loads are provided as proviral copies/cell. In the cats in group B, the FeLV provirus was detectable significantly earlier (in week 1) than in the cats in group A (p_Fisher_ = 0.0079). The cats in group B had significantly higher proviral loads in weeks 1 and 2 but lower loads in weeks 7–12 and 15 (p_MWU_ < 0.05; indicated by an *asterisk*). The plasma viral RNA loads are provided as copies/mL of plasma. The cats in group B had significantly higher viral RNA loads in week 1 but lower loads in weeks 4, and 7–11 (p_MWU_ ≤ 0.05; indicated by an *asterisk*). All cats in group C were negative at all tested time points (data not shown)

All of the recipient cats in groups A and B became FeLV-infected after blood transfusion as judged by proviral polymerase chain reaction (PCR) from peripheral blood and plasma viral RNA detected by reverse transcriptase (RT-)PCR (Fig. 1). By contrast, all of the cats in the control group C remained FeLV-negative throughout the study. The cats in group B (receiving blood with provirus and viral RNA) became FeLV provirus-positive significantly earlier than did the cats in group A (receiving blood with the provirus only): in week 1 post transfusion, all of the cats in group B were already provirus positive, whereas none of the cats in group A were positive (p_Fisher_ = 0.0079; Fig. 1a, b). In the first 2 weeks after the transfusion, the proviral loads were significantly higher in group B than in group A (Fig. 1a, b; p_MWU_ < 0.05). By contrast, in weeks 7–12 and 15, the proviral loads were significantly lower in the cats in group B than in those in group A (p_MWU_ < 0.05; Fig. 1a, b).

All of the recipient cats in groups A and B also turned plasma viral RNA-positive (Fig. 1c, d). The plasma viral RNA loads were significantly higher in the cats in group B compared with those in group A 1 week post transfusion (p_MWU_ = 0.0079; Fig. 1c, d). However, the cats in group B exhibited lower plasma viral RNA loads than did the cats in group A (significantly lower in weeks 4 and 7–11; p_MWU_ < 0.05). By week 15 post transfusion, three cats in group B exhibited undetectable plasma viral RNA loads, whereas all of the cats in group A remained positive.

**Fig. 2:**
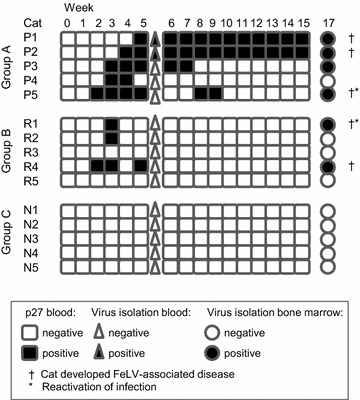
Outcome of FeLV infection in the recipient cats post transfusion. Results from p27 ELISA from the blood, virus isolation from the blood and virus isolation from the bone marrow of the five cats in group A, the five cats in group B and the five cats in the negative control group C. Four weeks post transfusion, significantly more cats were p27-positive in group A compared with group B (p_Fisher_ = 0.0476)

**Fig. 3:**
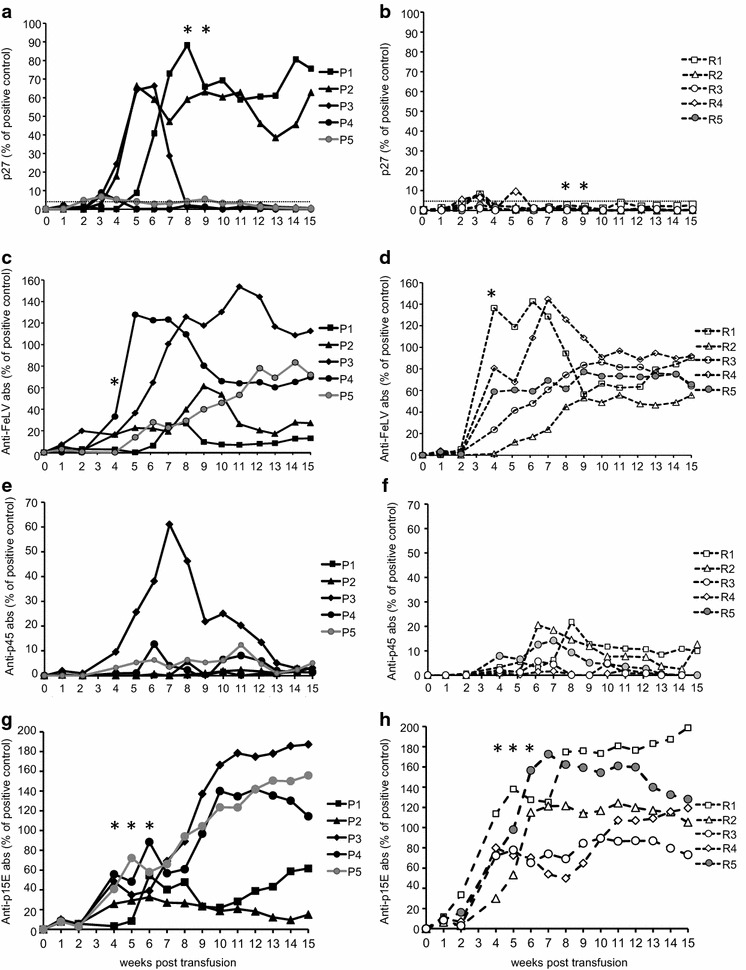
FeLV p27 antigen loads (**a**, **b**) and antibodies to FeLV of the five cats in group A (**a**, **c**, **e**, **g**) and the five cats in group B (**b**, **d**, **f**, **h**) total anti-FeLV antibodies (**c**, **d**), anti-p45 antibodies (**e**, **f**) and anti p15E antibodies (**g**, **h**). The p27 loads and the antibody levels are provided as percentages of a positive control run on each plate. Significant differences between groups A and B are indicated by an *asterisk*

Ethylenediaminetetraacetic acid (EDTA)-anticoagulated plasma samples were also analyzed for the presence of the FeLV p27 antigen as a marker for viremia by sandwich enzyme-linked immunosorbent assay (ELISA). All recipient cats in group A tested p27-positive at some time point after transfusion (Fig. 2). Two of the five cats in group A became persistently infected (progressive infection), and three cats developed a transient viremia and regressive infection (Fig. 3a). All of the five cats in group B developed regressive infection, three with transient viremia but low p27 values (max. 8 %) and two with undetectable antigenemia (Figs. 2, 3b). Four weeks post transfusion, significantly more cats were p27-positive in group A than in group B (p_Fisher_ = 0.0476; Fig. 2). When analyzing the p27 loads, the cats in group A exhibited significantly higher p27 loads in weeks 8 and 9 after transfusion than did the cats in group B (p_MWU_ = 0.0317; Fig. 3).

### Replicating virus, latent virus and viral shedding in recipient cats

Peripheral blood was collected in week 5 post transfusion and tested for the presence of replicating virus using virus isolation assays in cell culture. The two progressively infected cats in group A (cats P1, P2) tested positive (Fig. 2), whereas all of the other recipients tested negative. Moreover, bone marrow was collected 17 weeks post transfusion and tested in cell culture in the presence of a high dose of hydrocortisone for replicating virus. Latent bone marrow infection was detectable in six recipient cats: four in group A and two in group B (Fig. 2).

The shedding of FeLV viral RNA was investigated using RT-PCR in saliva samples collected at week 0 prior to the transfusion and in weeks 3, 5 and 7 post transfusion. Saliva viral RNA was detected in all recipient cats in groups A and B with particularly high RNA loads in the two cats with progressive infection, P1 and P2 (for details, see the table in Additional file 1: Table S1).

### Humoral immune response to FeLV

**Fig. 4:**
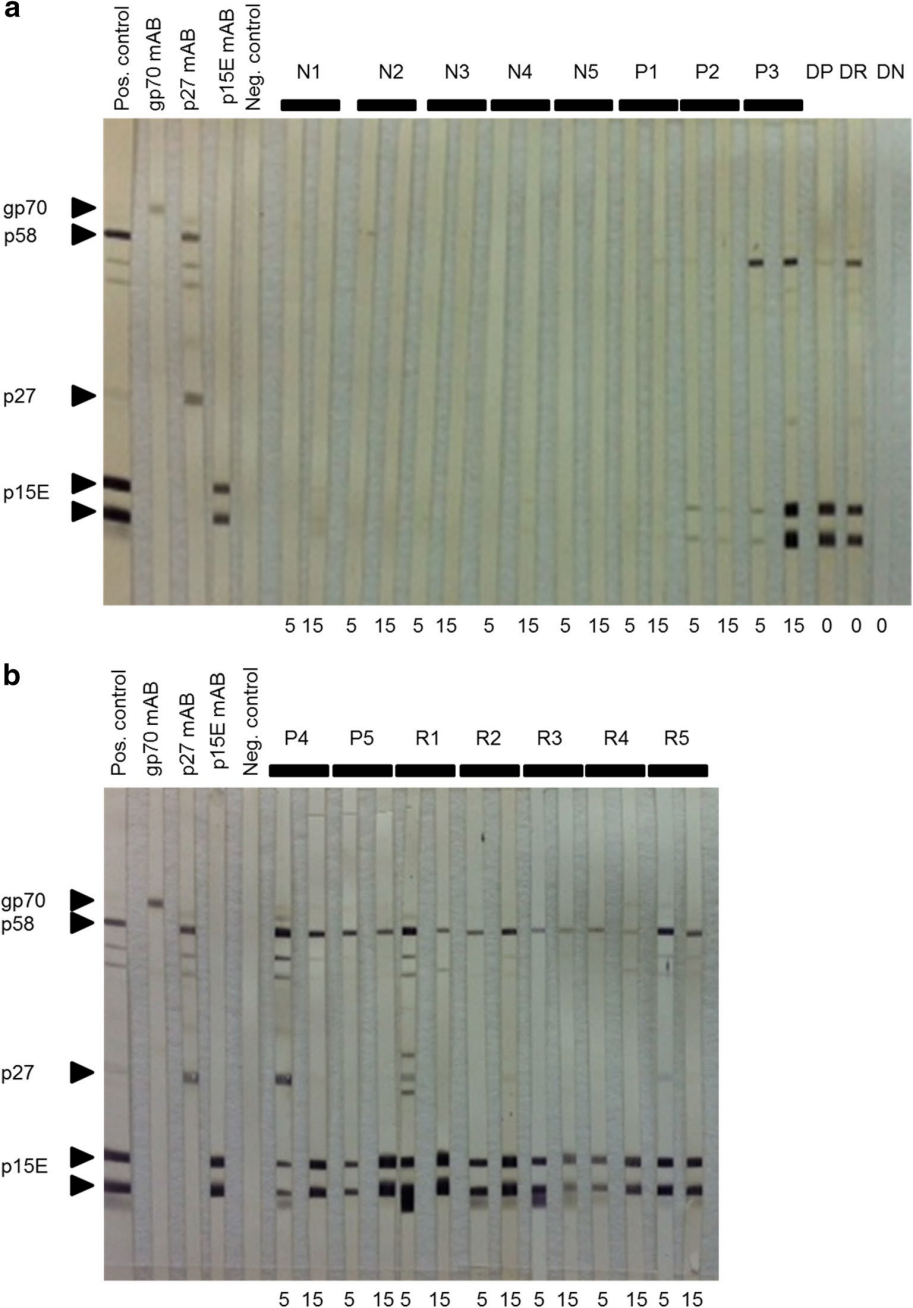
Western blot analysis using plasma samples from week 5 and 15 weeks post transfusion. The first five strips were incubated with a positive control (pool of immune cats), monoclonal antibodies against gp70, p27 and p15E, respectively, and a negative control (pool FeLV-negative cats) to characterize the respective viral proteins. **a** Cats N1 to N5, P1 to P3 and DP, DR and DN as indicated. **b** Cats P4 and P5 and R1 to R5 as indicated. *Right-pointing triangle* denotes bands with expected length. All samples were tested under the same assay conditions

Antibodies to FeLV were detected in plasma samples using three different ELISAs (Fig. 3): total anti-FeLV (whole virus), anti-p45 (unglycosylated envelope surface protein) and anti-p15E (transmembrane protein). All of the recipient cats of groups A and B developed antibodies to FeLV to some extent (Figs. 3, 4). The lowest antibody levels were observed in the two persistently infected cats P1 and P2 in group A (Fig. 3). The antibody response was earlier and higher in cats in group B than in cats in group A: in week 4 after transfusion, four of five cats in group B had total anti-FeLV antibody levels >20 % of the positive control, whereas only one of the five cats in group A surpassed 20 % in the whole virus antibody assay (Fig. 3c, d). Four out of the five cats in group B, but none of the cats in group A, presented antibodies to p15E >60 % of the positive control (Fig. 3g, h; p_Fisher_ = 0.0476). The cats in group B also exhibited significantly higher antibody levels to p15E than the cats in group A in weeks 5 and 6 post transfusion (p_MWU_ < 0.05). Antibodies to p15E remained low in the two persistently viremic cats, P1 and P2. Moreover, in cat P3, only transient antibodies to p45 were detectable at levels >25 % of the positive control (Fig. 3e). At the end of the observation period at week 15 post transfusion, the two persistently viremic cats in group A (P1 and P2) displayed low antibody levels to the whole virus and p15E, whereas all of the cats with regressive infection displayed high antibody levels (Fig. 3).

To further dissect and confirm the antibody response, the samples from all cats collected in weeks 5 and 15 and from the blood donor cats collected at the time of the blood collection for the transfusion were analyzed by western blot (Fig. 4). None of the cats displayed notable bands to gp70. Strong bands to p15E were confirmed in all cats from groups A and B except for the two persistently viremic cats, P1 and P2. In addition, p15E bands in cat P3 became only strong in week 15 after this cat had overcome viremia. Only a limited response to p27 was observed in some cats in groups A and B and in the blood donor cat DR at the time of blood collection for the transfusion. None of the cats in group C (N1–N5) or the donor cat DN presented antibodies to FeLV.

### Endogenous FeLV-like sequences in blood donors and recipient cats

Because endogenous FeLV-like sequences can be considered as a potential confounder in experimental FeLV infection, enFeLV provirus loads were determined in peripheral blood samples of the 15 recipients and the three blood donors collected prior to the blood transfusion using three quantitative PCR assays. The cats in group A tended to have somewhat lower enFeLV-env, enFeLV-U3-2 and total enFeLV-U3 loads than the cats in group B but not to an extent that was statistically significant (p_MWU_ enFeLV-env = 0.0556; p_MWU_ enFeLV-U3-2 = 0.1508; p_MWU_ total enFeLV-U3 = 0.0952). No significant differences were observed when the enFeLV loads of the recipients in groups A and B were compared with the control cats in group C and blood donor cats (P_KW_ > 0.05; for the enFeLV loads, please see the figure in Additional file 2: Figure S1).

### Cytokine response

**Fig. 5:**
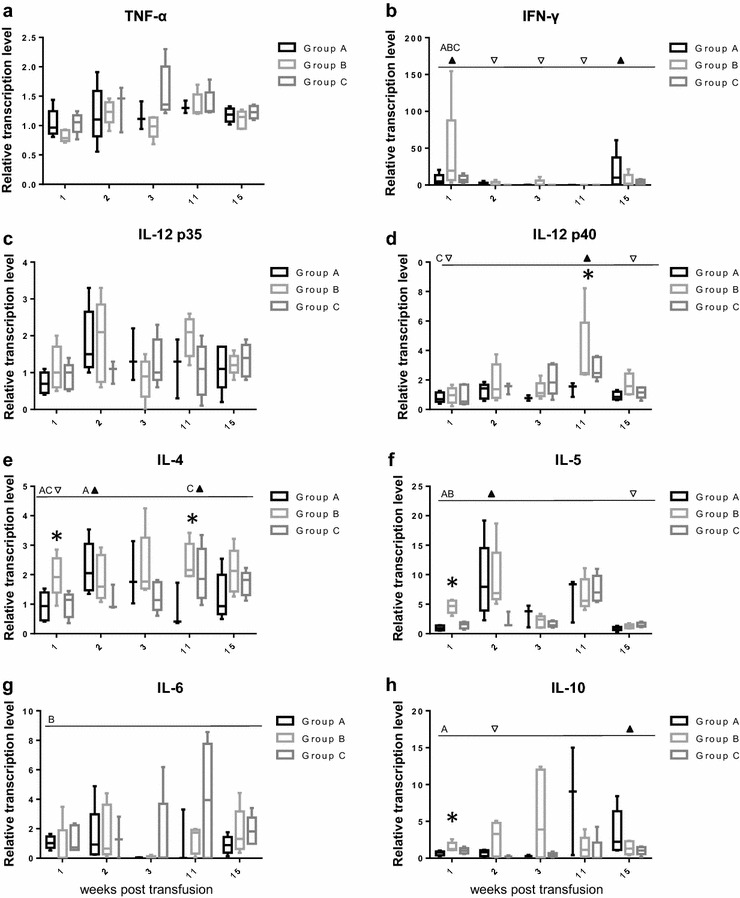
Transcription levels of cytokines after blood transfusion in the three groups of cats A, B and C **a** TNF-α, **b** IFN-γ, **c** IL-12 p35, **d** IL-12 p40, **e** IL-4, **f** IL-5, **g** IL-6 and **h** IL-10. The cytokine transcription levels were normalized to the transcription levels of two housekeeping genes (ABL and YWHAZ) at each time point and used to calculated relative transcription levels in relation to the levels in week 0 using GeNorm. Significant changes over time were tested by the Friedman test for each cytokine and each group, separately; significant differences are indicated by a *dotted line*; significantly higher (*upfilled-pointing triangle*) and lower (*down-pointing triangle*) time points in the Dunn’s posttest (P_D_) are denoted and the relevant group is listed (A, B or C). Significant differences between the three groups at one time point were tested using the Kruskal–Wallis test and are indicated by an *asterisk*

To characterize the immune response after the blood transfusion and FeLV infection in more detail, the transcription levels of cytokines were determined in EDTA-anticoagulated blood 0, 1, 2, 3, 11 and 15 weeks after the blood transfusion (Fig. 5). To assess a potential Th1 response, feline interferon-γ (IFN-γ) and interleukin (IL-12) were measured. To determine a potential Th2 response, IL-4, IL-5, IL-6 and IL-10 were also quantified. Tumor necrosis factor-α (TNF-α) and IL-6 were assessed as pro-inflammatory cytokines, whereas IL-10 exhibits anti-inflammatory effects. The most pronounced up-regulation was observed in IFN-γ levels (Th1) in the cats in group B (up to 155-times increase compared with the pre-transfusion level). The IFN-γ levels changed significantly over time for each of the three groups (A: p_F_ = 0.0239; B: p_F_ = 0.0053; C: p_F_ < 0.0001), with significantly higher levels in weeks 1 and 15 compared with those in weeks 2, 3 and 11 (Fig. 5b). Up to a ~20-fold increase was observed in IL-5 levels (Th2), with significant changes over time for all three groups (A: p_F_ = 0.0239; B: p_F_ = 0.0010; C: p_F_ = 0.0196) and significantly higher levels of IL-5 in groups A and B in week 2 compared with those in week 15 (Fig. 5f). A significant difference in IL-5 levels among the three groups was observed in week 1 (p_KW_ = 0.0004), with significantly higher IL-5 levels in group B than in group A (p_D_ < 0.01). Up to 15-fold up-regulation was observed for IL-10 (Th2 and anti-inflammatory) compared with the pre-transfusion levels; a significant change over time was observed for group A, with significantly higher levels in week 15 compared with those in week 2 (p_F_ = 0.0239; p_D_ < 0.05; Fig. 5h). A significant difference in IL-10 levels among the three groups was observed in week 1 (p_KW_ = 0.0176), with significantly higher IL-10 levels in group B compared with group A (p_D_ < 0.05). The maximum up-regulation in IL-6 and IL-12 p40 was 8- to ninefold, respectively (Fig. 5g, d). A significant change over time was observed for IL-12 p40 (Th1) in group C (p_F_ = 0.0055), with higher levels in week 11 compared with those in weeks 1 and 15 (p_D_ < 0.05); in week 11, a significant difference was observed among the three groups in IL-12 p40 levels (p_KW_ = 0.0273). For IL-6 (Th2 and pro-inflammatory), a significant change over time was observed for group B (p_F_ = 0.0244) but lost significance in the posttests. Significant changes over time were also observed for IL-4 (Th2) for groups A (p_F_ = 0.0085) and C (p_F_ = 0.0031), with significantly higher IL-4 levels in week 2 compared with week 1 for group A and significantly higher levels in week 11 compared with week 1 for group C (both p_D_ < 0.05; Fig. 5e). Significant differences among the groups were observed in week 1 (p_KW_ = 0.0440) and week 11 (p_KW_ = 0.0391), with significantly lower levels of IL-4 in group A compared with those in group B at both time points (p_D_ < 0.05). No significant differences between the three groups and no significant changes over time were observed in the IL-12 p35 transcription levels (Fig. 5c), and only minor up-regulation was observed for TNF-α (up to 2.3-fold; Fig. 5a).

### Clinical and laboratory findings

For 15 weeks post transfusion, weekly clinical examinations were performed by veterinarians. Two cats in group A (viremic P2 and P3) and one cat in group B (R1) exhibited elevated body temperatures after transfusion of 39.8 °C in week 6, 40.1 °C in week 8 and 39.6 °C in week 3, respectively. The two cats in group A presented with anorexia and mild lethargy during these episodes; after 2 days of treatment with meloxicam and intravenous fluids, the two cats recovered. Lymphadenomegaly of the mandibular, cervicalis superficialis and popliteal lymph nodes was observed in all five cats in group A and four of the five cats in group B. The lymphadenomegaly was more distinct and persistent in the cats in group A than in group B. None of the cats in group C developed elevated body temperatures or lymphadenomegaly (for additional details, please see the table in Additional file 3: Table S2).

A complete hematological was performed from EDTA-anticoagulated blood samples collected from all recipient cats prior to the blood transfusion and weekly thereafter until week 15. In addition, clinical chemistry was performed from sera at week 0 and at the appearance of clinical signs. In accordance with increasing age of the cats, the median PCV of the cats in groups B and C significantly increased between 5 and 6 % over time (B: p_F_ = 0.0002, week 2–15 p_D_ = 0.0354; C: p_F_ = 0.0003, week 0–15 p_D_ = 0.0402; see also Additional file 4: Figure S2). A decrease in PCV was observed in several cats in group A at the onset of antigenemia approximately at weeks 4–5 post transfusion. This decrease resulted in significantly lower PCV in group A compared with group C 7 weeks post transfusion (p_MWU_ = 0.0317).

The white blood cell (WBC) counts significantly changed over time in group B (p_F_ = 0.0008); following a severe decrease in the WBC counts in all cats between weeks 0 and 2 (lowest value observed: 5.9 × 10^3^/μL; reference range at this age 23.2 ± 3.36 × 10^3^/μL, [34]), a significant rebound was observed (week 2–6; p_D_ = 0.0184; see also Additional file 5: Figure S3). The slowest recovery of WBC counts was observed in cat R4, which also overcame antigenemia at the latest time point within group B. This temporary decrease resulted in significantly lower WBC counts in the cats in group B compared with those in the cats in the control group C in week 2 (p_MWU_ = 0.0397). In group A, WBC counts also decreased post transfusion but not in all cats simultaneously. In the transiently antigenemic cats P4 and P5, a rebound of WBC counts was observed starting in weeks 5–6, whereas only a limited and late increase in WBC counts (starting in week 9) was observed in the two cats with progressive infection, P1 and P2.

In neutrophil granulocyte counts, a significant change over time was observed for group A (p_F_ = 0.0163) but lost significance in the posttests (data not shown). The lowest neutrophil counts and severe intermittent neutropenia were observed in the persistently infected cat P2, with counts as low as 0.2 × 10^3^/µL (reference range at this age 11.0 ± 1.77 × 10^3^/μL, [34]).

Significant changes over time were observed in the lymphocyte counts in group B (p_F_ = 0.0013) but not in groups A or C (see also Additional file 6: Figure S4). All of the cats in group B exhibited a decrease in lymphocyte counts from weeks 0–1 post transfusion. A similar decrease was also observed 1 week later in the cats P3, P4, P5 in group A (week 1–2)—these three cats all overcame antigenemia similar to the cats in group B.

In weeks 8 and 9 post transfusion, moderate hyperproteinemia and moderate hypoalbuminemia were detected in cat P3 in group A immediately after the cat had overcome antigenemia (Fig. 2) and had developed high levels of antibodies to FeLV (Fig. 3c). Serum protein electrophoresis revealed moderate hyperbetaglobulinemia and severe polyclonal hypergammaglobulinemia, compatible with chronic immune stimulation (data not shown).

### Long-term follow up and clinical outcome of the recipient cats

After the initial observation period of 15 weeks after the blood transfusion, all cats remained in the facility, were regularly examined, and hematology, blood chemistry and p27 analyses were performed routinely until week 149.

In week 26 post transfusion, the cat R1 in group B developed a reactivation of the FeLV infection with p27 blood levels of 113 % (see also [23]) and had to be euthanized due to severe dyspnea (Table 1). Upon necropsy, a multicentric lymphoma of the thymus, the mesenteric lymph node, the liver and the bone marrow was observed with secondary lymphoblastic leukemia (WBC = 49 × 10^3^ cells/µL; 75 % lymphoblasts; see also Additional file 7: Figure S5). Histopathological and immunohistological examination revealed a T-cell lymphoma (CD3^+^ CD79a^−^).

In week 28 post transfusion, the cat R4 in group B was euthanized because of pleural effusion. Upon necropsy, a multicentric T-cell lymphoma (CD3^+^ CD79a^−^) of the thymus, kidney and lumbar lymph node was observed (Table 1). The cat tested p27-negative at the time of death.

In week 31, the persistently infected cat P1 in group A had to be euthanized because of severe normocytic-normochromic non-regenerative anemia and moderate leukopenia due to moderate neutropenia. At the time of euthanasia, the p27 antigen load was 114 %. FeLV-C, which is typically associated with the development of non-regenerative anemia in FeLV-infected cats [7], was detectable by subgroup PCR (see also Additional file 8: Figure S6). In addition, FeLV-B was detected, which arises in many cats with FeLV-A infection [3].

In week 84, the persistently viremic cat P2 had to be euthanized because of severe dyspnea caused by pleural effusion when the cat was vaccinated but not re-exposed to FeLV (listed as BZ3 in [35]) in a follow-up study. Cytological examination of the pleural effusion revealed numerous lymphatic blasts with a total nucleated cell count of 40.53 × 10^3^/µL. Additionally, the cat exhibited moderate lymphopenia, moderate hypoproteinemia, moderately increased alkaline phosphatase activity and slight hypochloremia. The p27-value in this cat increased in 4 weeks from 75 to 174 %. Necropsy revealed a multicentric T-cell lymphoma (CD3^+^ CD79a^−^) in the thymus, pericardium, tonsils and prescapular lymph nodes.

The cat P3 in group A, which exhibited hyperproteinemia and polyclonal hypergammaglobulinemia 8 weeks after infection, subsequently developed feline lower urinary tract disease (FLUTD) 94 weeks after transfusion and had to undergo intensive care outside of the barrier facility in the Small Animal Clinic at the Vetsuisse Faculty; the cat was placed privately after complete recovery.

In week 133 post transfusion, the cat P5 in group A had to be euthanized due to clinical deterioration (persistent anorexia, apathy). The cat exhibited anisocoria in week 131 and tested p27-positive (11 %), indicating reactivation of the infection. The p27 value increased subsequently to 27 % at the time of euthanasia. The samples collected prior to euthanasia were all viral RNA-positive. Upon necropsy, a multicentric T-cell lymphoma (CD3^+^ CD79a^−^) of the thymus and liver was observed (Table 1).

The remaining four of ten FeLV exposed recipient cats and all control cats in group C remained healthy to the time point of writing this manuscript (149 weeks after blood transfusion).

### Long-term follow up and clinical outcome of the blood donor cats

The blood donor cat for group B, cat DR, but not for group A, cat DP, was confirmed latently FeLV infected judged from bone marrow cell culture (in the presence of high doses of hydrocortisone) 18 weeks after the blood collection for the blood transfusion and 60 weeks after experimental FeLV exposure. At this time point, the cat DR demonstrated a reactivation of the FeLV infection with a p27 blood level of 132 % (see also [23] listed as GCN4). Clinically, the cat exhibited severe dyspnea and pleural effusion. Upon necropsy, a multicentric T-cell lymphoma (CD3^+^ CD79a^−^) of the thymus and inguinal lymph node was identified (Table 1). The blood donor for group A, cat DP, remained p27-negative and clinically healthy until 149 weeks after blood collection for the transfusion and 191 weeks after FeLV exposure.

## Discussion

Our results demonstrate for the first time that FeLV infection can be transmitted to naïve recipients via the transfusion of blood from a FeLV provirus carrier. Blood was collected for the transfusion 42 weeks after the blood donors had been exposed to FeLV and contained a low provirus load. Approximately 10^4^ provirus copies within 10 mL of blood were sufficient to result in a productive FeLV infection in all recipients. Young cats were selected for this experiment because they are more susceptible to FeLV infection than adult cats [36]. Although this increased susceptibility in young cats may be because of immaturity of the immune system, increased susceptibility to FeLV may also be expected in blood transfusion recipients because of severe clinical conditions and/or possible immunosuppression. Moreover, the transfused volume, 10 mL, was adjusted to the body weight of the kittens (~1–1.5 kg at the time of the transfusion) to reflect the volume of a regular blood transfusion in adult cats (usually 40–60 mL for a cat of ~4 kg body weight). Thus, the chosen experimental setup may well mirror the situation in the field for adult blood transfusion recipients. Some of the recipient cats subsequently developed FeLV-associated disease, confirming our hypothesis that whole blood containing white blood cells carrying FeLV provirus will transfer the virus and result in full-blown infection in recipient cats. Our results confirm and extend previous results in which FeLV infection was transmitted by subcutaneous injection of a plasmid carrying the proviral DNA [33]. Additionally, productive infection after intramuscular or intradermal injection of plasmids carrying proviral DNA were reported for the feline immunodeficiency virus in cats [37, 38] and the simian immunodeficiency virus in macaques [39]. Our study provides direct biological relevance and clinical importance: we conclude that there is an imminent risk of FeLV transmission via blood transfusion of provirus-positive blood, which must be considered in the clinic. By performing the commonly used blood donor screening assay (detecting the p27 antigen), both of the FeLV-infected blood donors that were used in the current study would not have been recognized as a risk. Therefore, we highly recommend screening all feline blood donors and blood products not only for FeLV antigen but also for FeLV provirus using sensitive PCR.

The provirus in the peripheral blood of the blood donors was replication-competent ~10 months after the initial virus exposure; however, viral replication was suppressed by the blood donors’ immune system to undetectable levels. When the cells containing the provirus entered a new susceptible host via blood transfusion, viral replication reignited. We were unable to determine from our data whether this reigniting was primarily because of the absence of a prepared immune system in the recipient cat or also because of a mixed lymphocyte-type reaction resulting in blastogenesis in the donor lymphocytes and FeLV expression contributing to the fast onset of viral replication. Such a reaction would be expected to occur within hours to a few days after the blood transfusion; no blood samples were collected at these time points, e.g., for lymphocyte isolation, flow cytometry analysis of lymphocyte populations or cytokine measurements. One week after transfusion (the earliest time point analyzed), an increase in total lymphocyte numbers was observed only in some cats in groups A and C; by contrast, all cats in group B exhibited a decrease in lymphocyte counts, which does not support the hypothesis of a mixed lymphocyte reaction. Therefore, if one assumes that FeLV was actively suppressed in the blood donors by the immune system but not in the recipients, the immune mechanism predominantly involved in the control of FeLV replication was not or was insufficiently transferable via blood transfusion. The latter would be true for the cellular immune response; it has been demonstrated previously that the cellular immune response plays an important role in suppressing viral replication in FeLV infections [40].

The replication competence of the provirus was further corroborated when one of the FeLV-exposed blood donors (DR) presented with a reactivation of the infection and the development of a multicentric lymphoma 4 months after the blood collection or the transfusion. The second provirus-positive cat serving as the blood donor for group A (cat DP) remained healthy and clinically unremarkable 149 weeks after the initiation of this study, although its blood had resulted in progressive infection in two recipient cats and FeLV-associated fatal disease in three recipients. Cat DP remains provirus-positive with a low provirus load. Further studies will be necessary to analyze the immune response in this and other provirus-positive long-term survivors that successfully keep the replication competent virus in check.

The FeLV infection that was observed in cats in groups A and B resembled a natural FeLV infection in regards to clinical findings, laboratory changes, infection outcome, disease spectrum and FeLV subgroup evolvement. The infected cats exhibited elevated body temperatures, lymphadenomegaly, anorexia, lethargy and changes in hematological parameters, including anemia and neutropenia. The severity of the clinical signs was associated with the severity of the infection: the lymphadenomegaly was particularly pronounced in the two viremic cats in group A, P1 and P2. Natural FeLV infection is characterized by leukopenia, lymphopenia and variable degrees of anemia [1]. A decrease in the WBC and lymphocyte counts was also observed in the present study in the FeLV-exposed cats but not in the control cats. The decrease in WBC and to some degree in lymphocyte counts appeared to be directly associated with the initial FeLV infection and the onset of viral replication; furthermore, the cats that recovered quickly from antigenemia exhibited a fast rebound of WBC counts. In addition, the cats in group A did not exhibit an adequate increase in PCV as observed in the control cats and related to the increasing age of the cats [34].

Replicating viruses were detected by cell culture in the blood or the bone marrow (the latter in the presence of high doses of hydrocortisone) in six recipient cats in groups A and B, confirming the replication capacity of FeLV in these six cats after infection via blood transfusion containing proviral DNA ± viral RNA. In addition, all eight of the recipient cats with regressive infection developed antibodies to FeLV, indicating the presence of at least minimal viral replication in these cats. By contrast, persistently infected cats exhibited lower antibody levels, as has been previously described in natural and experimental FeLV infected cats [19, 41]. Incidentally, no antibodies to gp70 were detectable in any of the cats by western blot. The virus preparation used for the blots were produced on FL-74 cells that harbor FeLV-ABC, but mainly C, for practical reasons (considerably higher yield), whereas the cats of the present study had been exposed to FeLV-A. gp70 but not p27 of FeLV-A and –C exhibited antigenetic differences. Moreover, gp70 may also be denatured during the antigen preparation (boiling in SDS), potentially resulting in loss of the three-dimensional structure and a lack of antibody binding. No samples were available for virus neutralization assays in the present study. Consistent with earlier data [41], only a few cats with regressive infection developed antibodies to p27. It is quite unlikely that these antibodies interfered with the detection of the p27 antigen in these cats; sera containing strong antibody reactivity to FeLV p27 did not interfere with antigen detection supposedly because of the high affinity of the anti-p27 monoclonal antibodies used in the p27 ELISA [42]. Subsequently, five of the ten recipient cats succumbed to FeLV-associated diseases. The cats suffered from non-regenerative anemia, T-cell lymphoma and leukemia, which are commonly regarded as FeLV-associated diseases [1]. Finally, the appearance of non-regenerative anemia was associated with the emergence of FeLV-C. This result also parallels natural FeLV-infection, in which the development of FeLV-C from FeLV-A via mutations has been described for FeLV-infected cats suffering from non-regenerative anemia [3, 43].

The cats that received blood containing only proviral DNA (group A) exhibited a later onset but graver outcome of FeLV infection compared with the cats that were transfused with blood containing not only proviral DNA but also viral RNA (group B). Initially, the cats in group B became provirus-positive significantly earlier after transfusion and developed significantly higher viral RNA and proviral loads than did the cats in group A. However, several weeks after the transfusion, the cats in group A exhibited significantly higher viral RNA, proviral and p27 antigen loads compared with those in group B. Moreover, more cats in group A were p27 antigen-positive compared with those in group B, two cats in group A but none in group B developed persistent infection, and some cats in group B but none in group A became viral RNA-negative within the observation period. A possible explanation for these observations could be the more efficient detection of FeLV by the immune system of cats in group B because of the presence of viral RNA and the higher proviral DNA loads in the transfused blood compared with those in group A. In group A, only low loads of provirus hidden within the donor cells were present. The differences in viral loads were paralleled by variations in the leukogram: a marked decrease in WBC and lymphocyte counts was observed in the cats in group B in week 1 when those cats, but not those of group A, tested viral RNA positive. Moreover, 1 week post transfusion in all cats, but particularly in the cats in group B, high IFN-ɣ levels with a maximum ~ 150-times increase were observed. IFN-γ plays a critical role in the innate and adaptive immunity against viral infections, partially through direct inhibitory effects on viral replication but primarily by immune stimulatory and modulatory effects on cellular defense mechanisms [44]. In FeLV-specific immunity, an important role of specific cytotoxic T lymphocytes was demonstrated, and FeLV-specific effector cytotoxic T-cell responses were detected as early as 1 week after virus exposure and were over the course of the early infection particularly high in cats that recovered from viremia [40]. Furthermore, the cats in group B exhibited higher IL-4, IL-5 and IL-10 levels compared with those in group A in week 1 after the transfusion; however, the changes in these cytokines were much less pronounced than in IFN-ɣ. IL-4 is a key regulator of humoral immunity and promotes the differentiation of Th2 cells, whereas IL-5 is produced by Th2 cells and stimulates B cells and antibody secretion. IL-4 and IL-5 levels increased in the cats in group A in week 2. Moderately increased levels of IL-10 were observed in group B around week 2 or 3, whereas in group A, these levels were detected only in weeks 11 and 15. IL-10 has multiple effects but is also associated with B cell proliferation and antibody production. Accordingly, more cats with high anti-FeLV antibody levels were observed in group B than in group A 4–6 weeks after virus exposure. Altogether, the data indicate that the immune systems of the cats in group B reacted quicker and more pronounced to the transfused blood and the virus that was transmitted with the blood compared with the cats in group A. This result supports our assumption of more efficient detection of FeLV by the recipients of the blood from blood donor B, in which viral RNA and higher proviral loads were present.

An additional factor resulting in differences in viral loads after FeLV exposure may have been the loads in enFeLV, which are present in each cell of the cat but with inter-individual differences. An association between enFeLV loads and FeLV replication has been reported previously: cats with high enFeLV loads exhibited higher viral replication than cats with low enFeLV loads in the early phase of a persistent FeLV infection [45]. Moreover, low enFeLV loads prior to FeLV exposure may be significantly associated with the development of a progressive infection, whereas cats with high enFeLV loads may rather develop a regressive infection [45, 46]. The cats in the present study originated all from the same SPF cattery, and the recipient cats were assigned into the three groups to avoid familial clustering. Nonetheless, the cats in group A tended to have somewhat lower enFeLV loads than the cats in group B, which would, as outlined above, fit to the lower FeLV replication observed early during infection and the higher number of cats who developed progressive infection in group A compared with group B.

A reactivation of FeLV infection was observed in three cats in the present study—one blood donor (cat DR, donor for group B) and two recipient cats (R1, group B and P5, group A)—further emphasizing the biological and epidemiological importance of provirus-positive cats and supporting our recommendation to test cats that are suspected to have been exposed to FeLV by PCR to identify FeLV provirus carriers. Subsequent to reactivation, provirus positive cats pose an infection risk to naïve cats. In addition, in our hands, all of the cats with reactivated infection developed fatal FeLV-associated disease [11, 22, 23]. Thus, to detect provirus carriers, e.g., to further decrease FeLV incidence in a feline population, cats with exposure risk should not only be tested for FeLV antigenemia/viremia but also for FeLV provirus. Plasma viral RNA could also be detected in the recipient cats R1 and P5 prior to reactivation, further supporting our hypothesis that the presence of plasma viral RNA may be associated with a higher probability of reactivation of the FeLV infection [11, 12]. Therefore, the detection of plasma viral RNA in provirus positive aviremic cats may serve as a marker for potential reactivation.

The recipient cats shed viral RNA in their saliva at varying degrees post transfusion. A high load of saliva viral RNA was primarily noted in the viremic cats, but FeLV RNA was also observed in cats with regressive infection, confirming a previous observation that during early infection, saliva viral RNA may not be a good marker for antigenemia/viremia [26], in contrast with long-term infected cats [28].

## Conclusions

Since the introduction of sensitive molecular methods, the occurrence of cats with FeLV proviral DNA in the absence of antigenemia and viremia has been well documented [10, 12, 19]. Up to 10 % of provirus-positive cats have been described among the Swiss cat population [19]. The results of our study underline the biological and epidemiological relevance of retroviral provirus in FeLV aviremic cats, further supporting observations that the proviral DNA in cats with regressive infection may maintain its replication capacity for many months to years [22, 23]. Sensitive PCR is recommended to detect provirus carriers as demonstrated in the current study to be performed in any cat serving as a blood donor to exclude inadvertent transmission of FeLV to the blood recipients but also in general to further decrease the incidence of FeLV infection within the cat population. The FeLV vaccines that are currently available can be efficacious in preventing persistent infection and the development of diseases subsequent to virus exposure; however, these vaccines do not prevent provirus integration [21]. Ideally, in the future, vaccines will be developed that also prevent provirus integration. Moreover, viral eradication treatments should be developed that eliminate proviral DNA in infected hosts. The latter is also a goal envisioned for HIV in humans [47, 48].

## Methods

### Animals

A total of 18 SPF cats (Liberty Research, Waverly, NY, USA) were included in this study: three adult male castrated SPF cats served as blood donors, and 15 eight-week-old-male SPF kittens served as blood transfusion recipients (Table 1). The recipient cats were randomly assigned to three groups of five cats each, avoiding familial clustering. Group A comprised cats P1–P5 (original cat IDs HBW2, HBZ3, HCC1, HCC4, and HCD1). Group B comprised cats R1–R5 (original cat IDs HBU1, HBW1, HBZ1, HCC3, and HBY1). Group C comprised cats N1–N5 (original cat IDs HBU2, HBV1, HBZ2, HCC2, and HCD2). Two of the three adult blood donors, DP and DR (original cat IDs GBX3, GCN4), had been experimentally exposed to FeLV-A/Glasgow-1 as previously described (listed as BX3 and CN4 in the previous study, [46]). These cats had undergone a regressive infection with undetectable antigenemia at the tested time points; the blood for the transfusion was collected 42 weeks after the infection. The third blood donor, DN (original cat ID GCN5), was a naïve adult SPF cat.

All cats were maintained in separated groups in a confined university facility under barrier conditions and optimal ethological and hygienic conditions as previously described [49]. All experiments were performed according to Swiss law and officially approved by the veterinary office of the canton of Zurich (160/2010, 129/2010). Prior to the start of the experiment, each cat was clinically examined, and blood and plasma samples and conjunctival, oropharyngeal, and rectal swabs were collected to verify the cats’ SPF status. These samples were tested for absence of FeLV, feline immunodeficiency virus, hemoplasmas, parvovirus, calicivirus, herpesvirus 1, coronavirus, *Bartonella henselae*, and *Chlamydophila felis*, as previously described [50]. In addition, the serum samples were tested for antibodies against feline calicivirus, feline herpesvirus and feline parvovirus by immunofluorescence assays as previously described [51]. All of the cats were negative for all of the tested infections. Upon arrival, the kittens were adapted to the new environment and the care persons for 2 weeks and trained for relaxed handling and blood collections without anesthesia.

### Experimental design and blood transfusion

For blood transfusion, 50 mL of blood was collected from each of the three adult blood donors—DP, DR and DN—under intramuscular anesthesia (10 mg/kg ketamine, Narketan^®^, Vétoquinol AG, Belp, Switzerland; 0.1 mg/kg midazolam, Dormicum^®^, Roche Pharma AG, Reinach, Switzerland). The blood was collected in a syringe containing 7 mL of the anticoagulant citrate phosphate dextrose adenine (CPDA-1, Fenwal, Lake Zurich, IL, USA). The blood from each donor was divided into five syringes of 10 mL—one syringe per recipient cat. The donor cats were administered 50 mL of Ringer-Lactate solution (Fresenius Kabi, Stans, Switzerland) and monitored until recovery from anesthesia.

The 15 kittens each received 10 mL of anti-coagulated whole blood at the age of 10 weeks. The blood for the cats P1–P5 in group A was FeLV provirus-positive (1 × 10^3^ copies/mL of blood) and plasma viral RNA- and p27-negative (donor DP). The blood for cats R1–R5 was provirus- (6.25 × 10^3^ copies/mL of blood) and plasma viral RNA-positive (1.95 × 10^5^ copies/mL of plasma) and p27-negative (donor DR). The blood for cats N1–N5 was FeLV provirus-, viral RNA- and p27-negative (donor DN). Prior to the transfusion, the blood types of each donor and recipient cat were determined using a commercial immunochromatographic test (Lab Test A + B, Alvedia, France). Moreover, a standard saline-agglutination crossmatching procedure was performed at 37 °C with blood from the recipients and the corresponding blood donors [52]. All of the cats were of blood type A, and the recipient cats were compatible with the donors in the crossmatch. The kittens were anesthetized intramuscularly as described above, and 10 mL of whole blood was collected for baseline analysis and to prevent circulatory volume overload. Thereafter, 10 mL of blood from the respective blood donor was transfused slowly over 30 min to each recipient cat. During and after the blood transfusion, the kittens were monitored for adverse reactions. The general conditions of the cats were monitored daily. The recipient cats were castrated 24 weeks after transfusion.

### Hematology, clinical chemistry and serum protein electrophoresis

WBC counts and complete hemograms were performed using a Sysmex XT-2000iV (Sysmex Corporation, Kobe, Japan) [53]. Blood smears were stained using an automated staining instrument (HemaTek, Siemens, Switzerland), and differential counts were performed microscopically. In the case of severe anemia, manual reticulocyte counts were performed using a standard method based on Brilliant Cresyl Blue- (SIGMA-ALDRICH, Steinheim, Germany) stained blood smears. Clinical chemistry was analyzed using a Cobas Integra 800 system (Roche Diagnostics, Rotkreuz, Switzerland) at week 0 and at the appearance of clinical signs. Serum biochemistry analysis included bilirubin, glucose, blood urea nitrogen, creatinine, protein, albumin, cholesterol, alkaline phosphatase, aspartate aminotransferase, alanine aminotransferase, lipase, sodium, chloride, potassium, phosphorus and calcium levels. Serum protein electrophoresis was performed in one cat with hyperproteinemia in week 8 and 9 (cat P3, group A) using a semi-automated agarose gel electrophoresis system as previously described [54].

### Nucleic acid extractions, complementary DNA synthesis and quantification of FeLV proviral and viral RNA loads

To determine proviral loads, DNA was extracted from 100 µL of buffy coat obtained from 1 mL of EDTA-anticoagulated blood using the QIAamp Blood Kit (Qiagen, Hombrechtikon, Switzerland) and 100 µL of elution buffer. To determine plasma viral RNA loads, total nucleic acid (TNA) was extracted from 200 µL of EDTA plasma using the MagNA Pure LC Total Nucleic Acid Isolation Kit (Roche Diagnostics) and 100 µL of elution buffer. To determine the viral RNA loads in the saliva, the swabs were processed using 200 μL of phosphate-buffered saline as previously described [28]. To determine the cytokine transcription levels, 100 μL of EDTA-anticoagulated blood was mixed immediately after blood collection with 300 μL mRNA lysis buffer (mRNA Isolation Kit I, Roche Diagnostics) and frozen as previously described [55]. mRNA was purified using the mRNA Isolation Kit I on a MagNA Pure LC instrument (Roche Diagnostics) and 25 µL of elution buffer [55]. First-strand complementary DNA (cDNA) was synthesized using the High-Capacity cDNA Reverse Transcription Kit (Applied Biosystems, Rotkreuz, Switzerland) in duplicate from each sample. In all of the extractions, negative controls consisting of phosphate buffered saline were extracted in parallel to monitor for cross-contamination.

FeLV provirus loads were quantified by TaqMan PCR targeting the unique region U3 of the long terminal repeat of FeLV as previously described [56] using 5 μl of TNA and the TaqMan Fast Universal PCR Master Mix (Applied Biosystems). This assay amplifies the three FeLV subgroups A, B, and C, but not enFeLV. The lower limit of detection of this assay is one provirus copy per reaction [56]. The thermocycling conditions were as follows: an initial denaturation of 20 s at 95 °C, followed by 45 cycles of 95 °C for 3 s and 60 °C for 45 s. Provirus loads were normalized to feline albumin copy numbers using TaqMan PCR as previously described [57]. The number of FeLV proviral copies in 10 mL of transfused blood was calculated using the extraction, elution and PCR volumes and the copies/PCR measured by PCR. TNA from plasma and saliva was analyzed by TaqMan RT-PCR targeting the same U3 region as the provirus PCR as previously described [56],using 5 μl of TNA and the SuperScript™ III Platinum^®^ One-Step Quantitative RT-PCR System (Invitrogen, Basel, Switzerland). The lower limit of detection of this assay is 180 viral RNA copies per reaction [56]. Plasma viral RNA loads were calculated as the number of copies per milliliter of blood. The number of viral RNA copies in 10 mL of transfused blood was calculated using the PCV and the number of copies/mL of plasma that was determined by RT-PCR. All of the PCR and RT-PCR assays were run on an ABI 7500 FAST Real-Time PCR System (Applied Biosystems); negative, positive and extraction controls were included in each run.

### Quantification of endogenous FeLV-like proviral loads

enFeLV provirus loads were determined in peripheral blood samples collected prior to the blood transfusion using quantitative real-time TaqMan PCR assays on an ABI 7500 FAST Real-Time PCR System (Applied Biosystems) as previously described [45, 58]. The following enFeLV targets were measured: enFeLV-U3-1, enFeLV-U3-2 and enFeLV-env. Total enFeLV-U3 copy numbers were calculated by addition of the enFeLV-U3-1 and enFeLV-U3-2 copy numbers. Provirus loads were normalized to feline albumin copy numbers using TaqMan PCR as previously described [57].

### Serological assays

EDTA-anticoagulated plasma samples were analyzed for the presence of p27 by double-antibody sandwich ELISA using monoclonal antibodies to three epitopic regions of p27 as previously described [59]. Antibodies to the FeLV whole virus were determined by ELISA [19, 21] using 0.2 µg FeLV per well purified by sucrose gradient from cell culture supernatant from FL-74 cells chronically infected with FeLV as previously described [41, 60]. Antibodies to the recombinant p15E transmembrane protein (25 μg/well) and the recombinant non-glycosylated form of the gp70 surface glycoprotein (p45; 0.2 μg/well) were measured by ELISA as previously described [61, 62]. The results were calculated as the percentages of a defined positive control run with each test; samples reaching >4 % of the positive control were considered to be positive in the p27 ELISA [19]. In addition, antibodies to FeLV were determined by western blotting using 20 μg of FeLV per blot purified by sucrose gradient from FL-74 cell culture supernatant as previously described [60]. EDTA-anticoagulated plasma samples were diluted 1:200 and the peroxidase conjugated goat-anti-cat antibodies (Jackson ImmunoResearch Laboratories, Inc., PA) were diluted 1:1000. Primary in-house monoclonal mouse ascites antibodies to gp70, p27, p15E [59, 63] diluted 1:400 and a secondary peroxidase conjugated goat-anti-mouse antibody (Jackson ImmunoResearch Laboratories) at a dilution of 1:1000 were used as positive controls. Moreover, pooled EDTA plasma samples from immune and SPF cats were used as positive and negative controls, respectively.

No serological analysis was possible because of lack of sample volume in week 3. No heparin plasma samples were available to test FeLV neutralizing activity in the donor or recipient cats.

### Virus isolation from peripheral blood and bone marrow

For virus isolation from peripheral blood, lithium heparin-anticoagulated samples were collected from the recipients 5 weeks post transfusion. Virus isolation was performed using 200 µL of lithium heparin plasma and QN10S cells as previously described [64]. The cells were monitored daily for cytopathic effects; at day 7, cell culture supernatant was collected for the quantification of the p27 antigen by ELISA.

For the detection of latent infection [16], bone marrow aspirates were collected from the proximal humerus from the recipient cats 17 weeks post transfusion and from the blood donors 18 weeks after the blood collection for transfusion (60 weeks into infection) under general anesthesia and analgesia as previously described [50]. The bone marrow was collected in 3.5 mL of RPMI 1640 cell culture medium (SIGMA-ALDRICH, Buchs, Switzerland) with 200 mM L-glutamine, 400 U/mL penicillin, 400 µg/mL streptomycin (Invitrogen) and 10 % FCS (BioConcept, Allschwil, Switzerland). Crandell feline kidney (CrFK) cells (20,000 cells/mL) were cultured in a 24-well plate (TPP Techno Plastic Products AG, Trasadingen, Switzerland) to 75 % confluence. These cells were confirmed to be FeLV-negative by PCR. The bone marrow cells were resuspended in medium and co-cultured with the CrFK cells. The cells with medium only were concurrently cultured on each plate. The cell culture medium contained the following: RPMI and Mc Coys 5A (1:1; SIGMA-ALDRICH), 200 mM L-glutamine, 400 U/mL penicillin, 400 µg/mL streptomycin, 10 % FCS and 1 µg/mL hydrocortisone (SIGMA-ALDRICH). The cultures were maintained for 12 weeks, and half of the medium was replaced every 3rd day, when the supernatant was collected for p27 ELISA and FeLV RT-PCR.

### Detection of FeLV subgroups

The development of non-regenerative anemia in FeLV-infected cats is typically associated with the emergence of FeLV-C [7]. Therefore, in one cat (P1) that had to be euthanized with non-regenerative anemia, the FeLV subgroups were investigated in the blood plasma and bone marrow, both of which were collected at the time of necropsy. The FeLV-A-specific primers RB59 and RB17, the FeLV-B-specific primers RB53 and RB17 and the FeLV-C-specific primers RB58 and RB47 were used in a conventional PCR as previously described [22, 65, 66].

### Cytokine transcription

To quantify cytokine levels in the peripheral blood, real-time PCR and primers and probes were used as previously described [67–69] with the exception of IL-5. For IL-5, the primers fIL-5_121f 5′-TGAATAGGCTGGTGGCAGAGA-3′, fIL-5_194r: 5′-CAGGTTCCCGTCGCCTATC-3′ and the probe fIL-5_143p: 5′-FAM-CTTGGCACTGCTCTCCACTCATCGAACT-TAMRA-3′ were designed; the reverse primer anneals over an exon–exon junction. The IL-5 reaction contained 0.9 µM forward and reverse primers and 0.25 µM probe. All of the cytokine assays were performed using the TaqMan Fast Universal PCR Master Mix (Applied Biosystems) with an initial denaturation at 95 °C for 20 s followed by 45 cycles 95 °C for 3 s and 60 °C for 30 s. The V-abl Abelson murine leukemia viral oncogene homolog (ABL) and zeta polypeptide (YWHAZ) transcription levels served as reference genes for normalization, as previously described [70]. The calculation of the relative transcription levels was performed using GeNorm version 3.5 [71]. For several samples, the transcription levels of IL-10 (total n = 5; weeks 2, 3, and 11), IL-6 (total n = 11; weeks 1, 3 and 11) and IFN-γ (total n = 29; weeks 2, 3, 11) were below the detection limit of the real-time PCR. No samples were available for cytokine measurements between weeks 3 and 11.

### Necropsy

Six of the cats (DR, R1, R4, P1, P2 and P5; Table 1) had to be euthanized for humane reasons and underwent necropsy and histopathological examination. Tissue sections from the lymphoma were tested to identify B and T cells using a CD3 T-cell marker (M7254, DAKO) and the B-cell marker for CD79 (M7051, DAKO; RB-90-13-P, Labvision, Thermo Fisher Scientific, Fremont, USA).

### Statistics

Statistical analyses were performed using GraphPad Prism for Windows, Version 4.03 (GraphPad software, San Diego, CA, USA). Changes over time (same group, different time points) were tested using the Wilcoxon signed rank test for paired samples (p_W_) or the Friedman test for multiple samples (p_F_) with Dunn’s posttest (p_D_). Multiplicity adjusted p-values for each comparison in a family of comparisons were computed. The frequencies were compared using the Fisher’s exact test (p_Fisher_). The differences between groups A and B were compared using the Mann–Whitney U-test (p_MWU_), and the differences between all three groups were tested using the Kruskal–Wallis test (p_KW_). A p-value <0.05 was considered to be statistically significant.

## Additional files

10.1186/s12977-015-0231-z Table detailing viral shedding in the saliva of recipient cats. Viral RNA loads detected by quantitative real-time RT-PCR in saliva collected from the recipient cats.
10.1186/s12977-015-0231-z Figure displaying enFeLV loads. enFeLV-env (a), Boxplots of enFeLV-U3-1 (b), enFeLV-U3-2 (c) and total enFeLV-U3 (d) loads. enFeLV loads are provided as copy numbers per feline albumin gene copy number.
10.1186/s12977-015-0231-z Table detailing the results of the clinical evaluation of the recipient cats (lymph node size). The size of the lymph nodes was assessed in the 15 recipient cats throughout the observation period.
10.1186/s12977-015-0231-z Figure displaying the packed cell volume (PCV) of recipient cats throughout the observation period. The PCVs of the five cats in group A (a), group B (b) and the negative control group C (c). A significant change (increase) over time in PCV was observed in groups B and C (p_F_); in week 7, the cats in group A exhibited significantly lower PCVs compared with the control cats in group C (marked by *; p_MWU_ = 0.0317).
10.1186/s12977-015-0231-z Figure displaying the WBC counts in recipient cats throughout the observation period. The WBC counts of the five cats in group A (a), group B (b) and the negative control group C (c). A significant change of WBC counts was observed in group B (p_F_) due to a significant increase from week 2 to 6 (indicated by *; p_D_ = 0.0184). In week 2, the cats in group B exhibited significantly lower WBC counts compared with the control cats in group C (indicated by filled diamond; p_MWU_ = 0.0397).
10.1186/s12977-015-0231-z Figure displaying the lymphocyte counts in the recipient cats throughout the observation period. The lymphocyte counts of the five cats in group A (a), group B (b) and the negative control group C (c). A significant change of lymphocyte counts was observed in group B (p_F_).
10.1186/s12977-015-0231-zPhoto of a blood smear from cat R1 (group B) with lymphoblastic leukemia at the time of necropsy. Lymphoblast cells are marked with an arrow. a) The picture displays a large lymphoblast with moderate amounts of basophilic cytoplasm and a large, round nucleus with fine chromatin patterns and several large, indistinct nucleoli. There is also a medium-sized lymphocyte with moderate amounts of pale basophilic cytoplasm and a round nucleus with a coarse chromatin pattern. b) The picture shows a medium-sized to large lymphoblast with small amounts of basophilic cytoplasm and a large, round nucleus with a fine chromatin pattern and two prominent round nucleoli.
10.1186/s12977-015-0231-z Results of the FeLV subgroup PCR of cat P1 at the time of necropsy. The FeLV subgroups were investigated in the bone marrow (1) and blood plasma (2), both of which were collected at the time of necropsy from cat P1. The expected sizes of the positive controls were 1071 bp for system A (FeLV-A), 857 bp for system B (FeLV-B) and 1754 bp for system C (FeLV-C). PCR products were analyzed on a 1.5% agarose gel. P = positive control; N = negative control; M = Marker: Fermentas SM0333 (Thermo Scientific, Waltham, Massachusetts).

## Abbreviations

ABL: V-abl Abelson murine leukemia viral oncogene homolog
cDNA: complementary DNA
CrFK: Crandell feline kidney
DNA: deoxyribonucleic acid
EDTA: Ethylenediaminetetraacetic acid
FeLV: feline leukemia virus
IFN-γ: feline interferon-γ
ELISA: enzyme-linked immunosorbent assay
enFeLV: endogenous FeLV-like sequences
ERV-DC: feline endogenous gammaretrovirus
IL: interleukin
PCR: polymerase chain reaction
PCV: packed cell volume
RT-PCR: reverse transcriptase PCR
SPF: specified pathogen-free
TNA: total nucleic acid
TNF-α: tumor necrosis factor-α
WBC: white blood cell
YWHAZ: zeta polypeptide
