# Supplementary material for: Retroviral DNA—the silent winner: blood transfusion containing latent feline leukemia provirus causes infection and disease in naïve recipient cats
Source: Retrovirology. 2015 Dec 21;12:105. doi: 10.1186/s12977-015-0231-z (PMC4687292; doi:10.1186/s12977-015-0231-z)
Supplement: Supplementary file 1 [file 12977_2015_231_MOESM1_ESM.docx]

Additional File 1: Table detailing viral shedding in the saliva of recipient cats. Viral RNA loads detected by quantitative real-time RT-PCR in saliva collected from the recipient cats.

		Viral RNA loads in saliva (copies per RT-PCR)			
**Group**	**Cat**	**Week 0**	**Week 3**	**Week 5**	**Week 7**
**A**	P1	0	0	321	48 632
	P2	0	2	8	76 074
	P3	0	4	2	1 143
	P4	0	0	15	150
	P5	0	143	858	0
**B**	R1	0	17	23	22
	R2	0	8	16	8
	R3	0	0	4 714	17
	R4	0	2	12	226
	R5	0	3	0	2
**C**	N1	0	0	0	0
	N2	0	0	0	0
	N3	0	0	0	0
	N4	0	0	0	0
	N5	0	0	0	0
