# Supplementary material for: Retroviral DNA—the silent winner: blood transfusion containing latent feline leukemia provirus causes infection and disease in naïve recipient cats
Source: Retrovirology. 2015 Dec 21;12:105. doi: 10.1186/s12977-015-0231-z (PMC4687292; doi:10.1186/s12977-015-0231-z)
Supplement: Supplementary file 2 [file 12977_2015_231_MOESM2_ESM.docx]

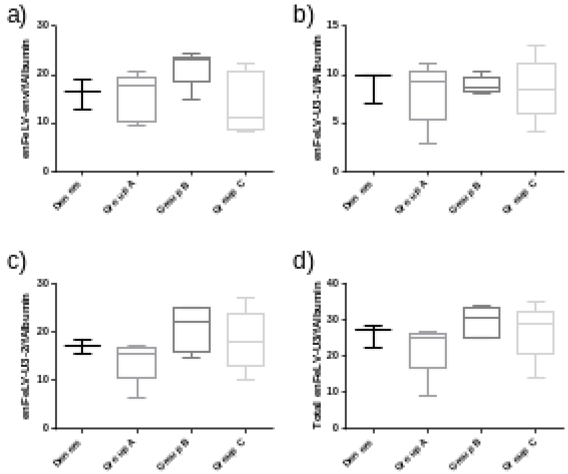

Additional File 2: Figure displaying enFeLV loads. enFeLV-env (a), Boxplots of enFeLV-U3-1 (b), enFeLV-U3-2 (c) and total enFeLV-U3 (d) loads. enFeLV loads are provided as copy numbers per feline albumin gene copy number.
