# Supplementary material for: Retroviral DNA—the silent winner: blood transfusion containing latent feline leukemia provirus causes infection and disease in naïve recipient cats
Source: Retrovirology. 2015 Dec 21;12:105. doi: 10.1186/s12977-015-0231-z (PMC4687292; doi:10.1186/s12977-015-0231-z)
Supplement: Supplementary file 3 [file 12977_2015_231_MOESM3_ESM.docx]

Additional File 3: Table detailing the results of the clinical evaluation of the recipient cats (lymph node size).

The size of the lymph nodes was assessed in the 15 recipient cats throughout the observation period

Group	Cat	Weeks															
		0	1	2	3	4	5	6	7	8	9	10	11	12	13	14	15
**A**	P1	-	-	-	-	-	-	++	++	+++	++	++	++	++	++	+	+
	P2	-	-	-	-	-	+	+	+++	++	++	+	+	+	+	+	-
	P3	-	-	-	-	-	++	+++	++++	++++	+++	+++	++	++	++	+	+
	P4	-	-	-	-	-	++	++	+	-	-	-	-	-	-	-	-
	P5	-	-	-	-	-	+	-	-	-	-	-	-	-	-	-	-
**B**	R1	-	-	-	-	-	+++	+	+	+	+	+	-	-	-	-	-
	R2	-	-	-	-	-	+	-	-	-	-	-	-	-	-	-	-
	R3	-	-	-	-	-	+	-	-	-	-	-	-	-	-	-	-
	R4	-	-	-	-	-	++	+++	++	+	+	+	+	-	-	-	-
	R5	-	-	-	-	-	-	-	-	-	-	-	-	-	-	-	-
**C**	N1	-	-	-	-	-	-	-	-	-	-	-	-	-	-	-	-
	N2	-	-	-	-	-	-	-	-	-	-	-	-	-	-	-	-
	N3	-	-	-	-	-	-	-	-	-	-	-	-	-	-	-	-
	N4	-	-	-	-	-	-	-	-	-	-	-	-	-	-	-	-
	N5	-	-	-	-	-	-	-	-	-	-	-	-	-	-	-	-

The size (diameter) of the mandibular, cervicalis superficialis and popliteal lymph nodes were determined by palpation, the mean diameter calculated and categorized as follows: normal lymph node size = -, 1 cm diameter = +, 2 cm diameter = ++, 3 cm diameter = +++, = 4 cm diameter = ++++.
