# Supplementary material for: Retroviral DNA—the silent winner: blood transfusion containing latent feline leukemia provirus causes infection and disease in naïve recipient cats
Source: Retrovirology. 2015 Dec 21;12:105. doi: 10.1186/s12977-015-0231-z (PMC4687292; doi:10.1186/s12977-015-0231-z)
Supplement: Supplementary file 4 [file 12977_2015_231_MOESM4_ESM.docx]

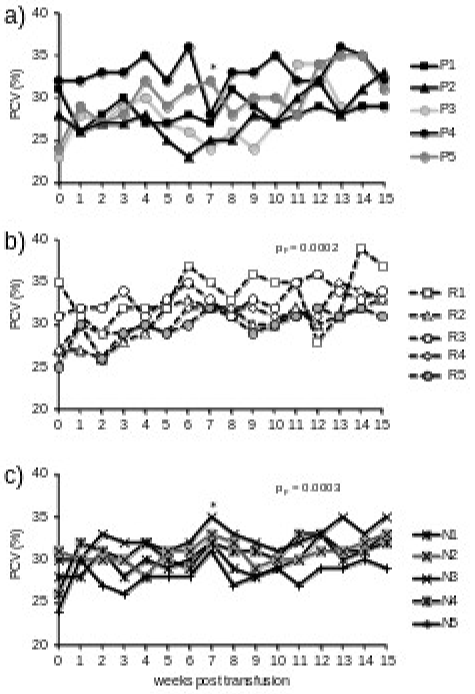

Additional File 4: Figure displaying the packed cell volume (PCV) of recipient cats throughout the observation period. The PCVs of the five cats in group A (a), group B (b) and the negative control group C (c). A significant change (increase) over time in PCV was observed in groups B and C (p_F); in week 7, the cats in group A exhibited significantly lower PCVs compared with the control cats in group C (marked by *; p_{MWU} = 0.0317).
