# Supplementary material for: Retroviral DNA—the silent winner: blood transfusion containing latent feline leukemia provirus causes infection and disease in naïve recipient cats
Source: Retrovirology. 2015 Dec 21;12:105. doi: 10.1186/s12977-015-0231-z (PMC4687292; doi:10.1186/s12977-015-0231-z)
Supplement: Supplementary file 5 [file 12977_2015_231_MOESM5_ESM.docx]

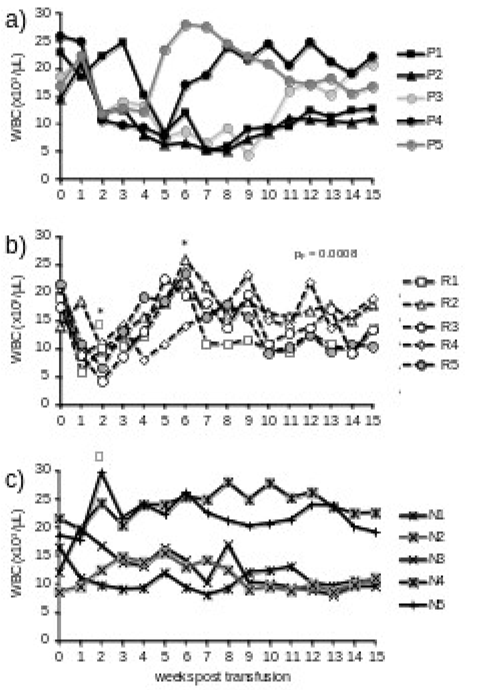

Additional File 5: Figure displaying the WBC counts in recipient cats throughout the observation period. The WBC counts of the five cats in group A (a), group B (b) and the negative control group C (c). A significant change of WBC counts was observed in group B (p_F) due to a significant increase from week 2 to 6 (indicated by *; p_D = 0.0184). In week 2, the cats in group B exhibited significantly lower WBC counts compared with the control cats in group C (indicated by ⧫; p_{MWU} = 0.0397).
