# Supplementary material for: Retroviral DNA—the silent winner: blood transfusion containing latent feline leukemia provirus causes infection and disease in naïve recipient cats
Source: Retrovirology. 2015 Dec 21;12:105. doi: 10.1186/s12977-015-0231-z (PMC4687292; doi:10.1186/s12977-015-0231-z)
Supplement: Supplementary file 6 [file 12977_2015_231_MOESM6_ESM.docx]

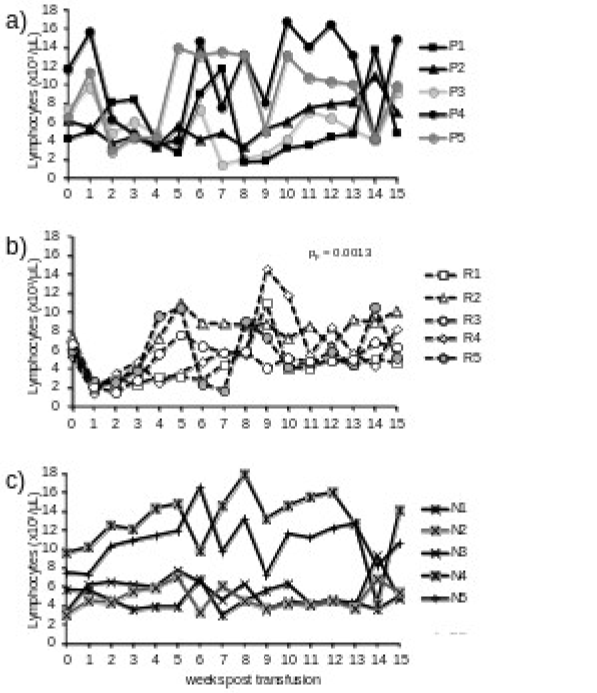

Additional File 6: Figure displaying the lymphocyte counts in the recipient cats throughout the observation period. The lymphocyte counts of the five cats in group A (a), group B (b) and the negative control group C (c). A significant change of lymphocyte counts was observed in group B (p_r).
