# Supplementary material for: Retroviral DNA—the silent winner: blood transfusion containing latent feline leukemia provirus causes infection and disease in naïve recipient cats
Source: Retrovirology. 2015 Dec 21;12:105. doi: 10.1186/s12977-015-0231-z (PMC4687292; doi:10.1186/s12977-015-0231-z)
Supplement: Supplementary file 7 [file 12977_2015_231_MOESM7_ESM.docx]

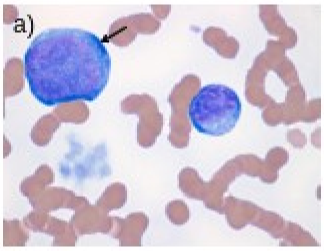

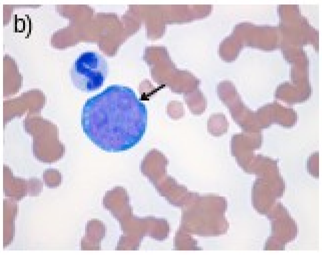

Additional File 7: Photo of a blood smear from cat R1 (group B) with lymphoblastic leukemia at the time of necropsy. Lymphoblast cells are marked with an arrow. a) The picture displays a large lymphoblast with moderate amounts of basophilic cytoplasm and a large, round nucleus with fine chromatin patterns and several large, indistinct nucleoli. There is also a medium-sized lymphocyte with moderate amounts of pale basophilic cytoplasm and a round nucleus with a coarse chromatin pattern. b) The picture shows a medium-sized to large lymphoblast with small amounts of basophilic cytoplasm and a large, round nucleus with a fine chromatin pattern and two prominent round nucleoli.
