# Supplementary material for: Retroviral DNA—the silent winner: blood transfusion containing latent feline leukemia provirus causes infection and disease in naïve recipient cats
Source: Retrovirology. 2015 Dec 21;12:105. doi: 10.1186/s12977-015-0231-z (PMC4687292; doi:10.1186/s12977-015-0231-z)
Supplement: Supplementary file 8 [file 12977_2015_231_MOESM8_ESM.docx]

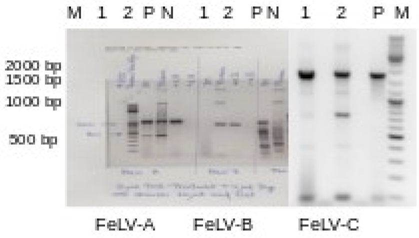

Additional File 8: Results of the FeLV subgroup PCR of cat P1 at the time of necropsy. The FeLV subgroups were investigated in the bone marrow (1) and blood plasma (2), both of which were collected at the time of necropsy from cat P1. The expected sizes of the positive controls were 1071 bp for system A (FeLV-A), 857 bp for system B (FeLV-B) and 1754 bp for system C (FeLV-C). PCR products were analyzed on a 1.5% agarose gel. P = positive control; N = negative control; M = Marker: Fermentas SM0333 (Thermo Scientific, Waltham, Massachusetts).
